# Settling Questions

**Published:** 1883-05

**Authors:** 


### Settling Questions.

Dr. Barrett says, in the March No. of the *Practitioner*: "Last month we published a paper by Dr. Stockwell on the 'Etiology of Dental Caries.' This month, we give another by Dr. Clark, both in support of the germ theory of decay. Next month, we shall print one by Prof. F. Abbott, in support of the vital theory, or the inflammatory hypothesis. Our May number will contain yet another paper by Dr. W. D. Miller, of Berlin, presenting his theory of dental caries. The June number will contain an article giving the views of those who support the chemical theory, prepared for us by one who is an acknowledged authority."

Doubtless, the profession will look with hopeful anticipation for these papers, and will peruse them with interest, and it is to be hoped with profit.

Much of the discussion on the subject of dental caries seems to be, either for the purpose of showing the writer's knowledge of technicalities, or to support some preconceived theory, neither of which aids much in the development and progress of science. It is unfortunate that those who are working in obscure and uncertain fields become unduly positive and intolerant of the opinions of others. How often is it, that a course of investigation of a few months, or two or three years at most, renders the investigator wholly incapable of even considering well established facts that have been developed by many long years of study, experiment and direct contact and observation, when all surrounding conditions and circumstances have been taken into account.

If those who profess to be making thorough investigation would pursue their research with the aim of ascertaining facts rather than to build a theory based only upon probabilities or possibilities, there would be more hope of progress.

Would it not be well to find some common ground upon which all can stand, some positions that have been clearly and fully established, and let this be a starting point for further ascertainments and advance; and let that which is written be stated in language that shall be clear and understandable by those who are expected to read.

We will look for the promised articles with interest and hopeful anticipation.

